# The science behind safety in our daily life: the open symposium of the Japanese environmental mutagen and genome society (JEMS), 2025

**DOI:** 10.1186/s41021-025-00346-8

**Published:** 2025-11-18

**Authors:** Naoki Koyama, Masataka Tsuda

**Affiliations:** 1 Translational Research Division, Safety and Bioscience Research Department, Chugai Pharmaceutical Co., Ltd, Yokohama, Kanagawa Japan; 2 Division of Genome Safety Science, National Institute of Health Sciences, Kawasaki, Kanagawa Japan

**Keywords:** Safety of pharmaceuticals and foods, Environmental exposure and health risks, PFAS, Radioactive substances, Formaldehyde health effects, Colibactin

## Abstract

The open symposium of the Japanese Environmental Mutagen and Genome Society (JEMS) entitled “The Science Behind Safety in Our Daily Lives,” was held as a hybrid in-person and online meeting on June 14, 2025. The rapid advancement of science and technology continues to profoundly alter our lifestyles. We face potential risks from chemical, biological, and physical agents, including chemical substances, bacteria/viruses, and radioactive substances, particularly in pharmaceuticals, food, and indoor environments. Furthermore, natural disasters such as earthquakes and heavy rains not only cause physical damage, but can also lead to health hazards from chemical substances and radiation. This underscores the urgent need for robust systems that can effectively respond to health crises. This symposium aimed to improve public understanding of safety science in daily life, including in pharmaceuticals, food, and living environments. In this symposium, we invited five scientists who are expanding the frontiers of health sciences. We organized this public event to be open to everyone, not just members of the JEMS. Herein, the organizers present a summary of the symposium.

## Background

The Open Symposium of the Japanese Environmental Mutagen and Genome Society (JEMS) is organized annually to present the cutting-edge technologies and science related to genetic toxicology and environmental mutagenesis to the public, with proceedings summarized in meeting reports [1–7]. Last year’s symposium, entitled “The Cutting Edge of Research on Protein/nucleic Acid Adduct Science” was organized by Dr. Yukari Totsuka, Dr. Yuji Ishii, and Dr. Koji Uchida. In 2025, the symposium, “The Science Behind Safety in Our Daily Lives” featured presentations by five scientists, including both JEMS and non-JEMS members. Our daily lives involve constant exposure to pharmaceuticals, food, and indoor environments that are increasingly susceptible to potential hazards from chemical, biological, and physical agents, including various chemical substances, bacteria, viruses, and radioactive substances. These challenges highlight the urgent need for robust, effective systems that can swiftly and comprehensively respond to health crises. The symposium aimed to bridge the gap between complex scientific knowledge and public understanding by raising awareness of essential safety measures related to pharmaceuticals, food, and our immediate living environments. The symposium was held on June 14th, 2025, in a hybrid format, accommodating both in-person attendees and those joining online. Through this report, the organizers present a summary of the event and its presentations.

## Symposium venue

Shimadzu Tokyo Innovation Plaza (Kawasaki, Kanagawa, Japan) with remote attendance via Microsoft Teams.

### Symposium program

Tomonari Matsuda (President, Japanese Environmental Mutagen and Genome Society; Kyoto University): Inaugural speech.

Naoki Koyama (Chugai Pharmaceutical): Introduction.

Session 1: Safety of Pharmaceuticals and Foods (Chair: Masataka Tsuda)

- Masamitsu Honma (National Institute of Health Sciences): Is it safe to eat? Safety of chemical substances in pharmaceuticals and foods.
- Akihiko Hirose (Chemicals Evaluation and Research Institute): Health effects of PFAS (Per- and Polyfluoroalkyl Substances).
- Tomoaki Tsutsumi (Division of Foods, National Institute of Health Sciences): Radioactive substances and food safety: Initiatives and current status after the Fukushima Daiichi Nuclear Power Plant accident.

Session 2: Environmental Exposure and Health Risks (Chair: Naoki Koyama)

- Kenichi Azuma (Faculty of Medicine, Kindai University): Health effects of formaldehyde in indoor and ambient air.
- Kenji Watanabe (School of Pharmaceutical Sciences, University of Shizuoka): Colibactin, a genotoxin produced by E. coli, and colorectal cancer.

Masataka Tsuda (National Institute of Health Sciences): Concluding speech.

## Meeting report

Dr. Masamitsu Honma presented on the safety of chemical substances found in pharmaceuticals and foods. He categorized carcinogens into two main types: genotoxic and non-genotoxic. Genotoxic carcinogens can induce DNA damage and mutations even at very low doses, whereas non-genotoxic carcinogens exhibit threshold-dependent risks, allowing for the establishment of acceptable exposure levels. He explained essential risk assessment concepts, including dose–response relationships and threshold values, using quantitative indices such as TD_50_ (tumorigenic dose rate 50) and virtually safe dose (VSD). Through illustrative case studies, he examined historical and naturally occurring substances: AF-2 (a banned food additive), arsenic (found in rice and hijiki), benzo[*a*]pyrene (in smoked foods), and acrylamide (formed during high-temperature cooking). These examples demonstrated how carcinogenic potency and actual human exposure levels differ, effectively highlighting the contrast between scientifically assessed risks and public perception. This underscores the need for evidence-based risk communication. Dr. Honma also discussed recent regulatory initiatives targeting genotoxic impurities in pharmaceuticals, with particular focus on nitrosamines. In alignment with international guidelines such as ICH-M7, he introduced the Carcinogenic Potency Categorization Approach (CPCA), a structure-based framework that enables more refined classification of nitrosamines and supports the setting of substance-specific acceptable intake limits. He concluded by emphasizing that while that zero risk is unattainable, science-based regulation grounded in quantitative thresholds plays a vital role in protecting public health. He stressed the importance of effective risk communication to help the public understand these scientific principles and to alleviate growing concerns over chemical safety in daily life.

Dr. Akihiko Hirose focused on the health effects of per- and polyfluoroalkyl substances (PFAS), particularly those relevant to environmental exposure. He explained that PFAS is a collective term for various organofluorine compounds, and that multiple definitions exist for identifying PFAS. According to the OECD’s 2018 definition, over 4,730 PFAS-related compounds have been identified, but following revisions in 2021, it is now estimated that over 10,000 PFAS-related compounds fall under this category. Due to their water- and oil-repellent properties and high physical and chemical stability, PFAS are widely used in a variety of applications, including solvents, surfactants, water- and stain-resistant coatings for textiles, leather, and paper, ion-exchange membranes, lubricants, firefighting foams, semiconductor production, and as processing aids for fluoropolymers. Regarding genotoxicity, he noted that PFOS, PFOA, and PFHxS do not exhibit direct genotoxicity, although some studies have shown that they can cause secondary DNA damage through oxidative stress. Therefore, the threshold approach is considered appropriate for evaluating the tolerable daily intakes of these PFAS compounds. Dr. Hirose also presented toxicological and epidemiological data indicating potential adverse health effects associated with PFAS exposure, including liver toxicity, thyroid disruption, immunotoxicity, and developmental and reproductive effects. He summarized recent regulatory trends in Japan and other countries, and stressed the importance of science-based risk assessment and effective public communication in addressing PFAS-related concerns.

Dr. Tomoaki Tsutsumi gave a talk on radioactive substances and food safety, focusing on the regulatory initiatives and current status following the Fukushima Daiichi nuclear power plant accident. The accident, triggered by the Great East Japan Earthquake in March 2011, led to widespread contamination of food with radioactive substances, presenting a major challenge to food hygiene and public health. In response, provisional regulatory values were promptly introduced, and in April 2012, the present standard limits for radioactive substances in food were established under the Food Sanitation Act. Dr. Tsutsumi provided an overview of the current regulatory framework for radioactive substances in food and presented recent data on radioactive cesium levels, internal radiation dose estimates from dietary intake, and trends in radioactive cesium (cesium-134 and cesium-137) and strontium-90. He also emphasized Japan’s ongoing efforts to ensure food safety through continuous monitoring, risk management, and transparent communication with the public.

Dr. Kenichi Azuma presented on the health effects of formaldehyde in indoor and ambient air. Formaldehyde has been widely used in disinfectants, wood adhesives, paints, and molded products. In particular, it is identified as a representative causative agent of “sick building syndrome” because urea resins used as adhesives in plywood for flooring, wall materials, and furniture hydrolyze, releasing formaldehyde into the air and contaminating indoor environments. While formaldehyde had shown positive results in numerous genotoxicity tests, suggesting its genotoxic potential, he highlighted that its toxic effects on tissues at contact sites were non-linear and highly concentration-dependent. This characteristic suggested the possibility of establishing a threshold for low-concentration exposure.

Dr. Kenji Watanabe presented on colibactin, a genotoxin produced by certain bacteria in the gut microbiota, and its role in colorectal cancer. Colibactin is identified as a genotoxic substance that induces DNA damage in mammalian cells, strongly implicating it as a causative agent of colorectal cancer. Although its structure was initially unknown due to instability, it has recently been elucidated. Substantial experimental evidence supports its carcinogenic role, including the detection of colibactin-DNA adducts and the discovery of specific mutation signatures induced by colibactin-producing bacteria in human colorectal cancer cell genomes. To prevent colibactin-induced cancer, Dr. Watanabe developed an IgM monoclonal antibody (*E. coli*-50 specific) for the rapid detection of colibactin-producing bacteria. Its epitope was identified as the *E. coli* O antigen, specifically the lipopolysaccharide’s sugar chain. An IgG monoclonal antibody was subsequently generated, and he reported on its specificity and its epitope’s glycan unit chemical structure, enabling immunostaining and neutralizing antibody production.

This symposium successfully brought together approximately 100 participants to explore critical health-risk issues. It was held in a hybrid format, both in-person and via Microsoft Teams, allowing active engagement from a wide range of attendees. Notably, 40% of the attendees were non-JEMS members, including members of the general public, which illustrates the success of our outreach efforts to promote public awareness of everyday safety science. As the organizers, we extend our sincere gratitude to all who participated in this symposium.

## Data Availability

Not applicable.

## Abbreviations

JEMS: The Japanese Environmental Mutagen and Genome Society
CPCA: Carcinogenic potency categorization approach
TD_50_: Tumorigenic dose rate 50
VSD: Virtually safe dose
PFAS: Poly- and perfluoroalkyl substances
PFOS: Perfluorooctane sulfonic acid
PFOA: Perfluorooctanoic acid
PFHxS: Perfluorohexanesulfonic acid
ELISA: Enzyme-Linked Immunosorbent Assay
IgM: Immunoglobulin M
IgG: Immunoglobulin G
